# Effects of roniciclib in preclinical models of anaplastic thyroid cancer

**DOI:** 10.18632/oncotarget.19092

**Published:** 2017-07-08

**Authors:** Shu-Fu Lin, Jen-Der Lin, Chuen Hsueh, Ting-Chao Chou, Richard J. Wong

**Affiliations:** ^1^ Department of Internal Medicine, Chang Gung Memorial Hospital, Chang Gung University, Taoyuan, Taiwan; ^2^ Department of Pathology, Chang Gung Memorial Hospital, Chang Gung University, Taoyuan, Taiwan; ^3^ Laboratory of Preclinical Pharmacology Core, Memorial Sloan Kettering Cancer Center, New York, NY, USA; ^4^ Current address: PD Science, Inc., Paramus, NJ, USA; ^5^ Department of Surgery, Memorial Sloan Kettering Cancer Center, New York, NY, USA

**Keywords:** roniciclib, cyclin-dependent kinase, anaplastic thyroid cancer

## Abstract

Many human cancers have altered cyclin-dependent kinase activity. Inhibition of cyclin-dependent kinases may arrest cell cycle progression and represents an important strategy in the treatment of malignancies. We evaluated the therapeutic effects of roniciclib, a cyclin-dependent kinase inhibitor, as a treatment for anaplastic thyroid cancer. Roniciclib inhibited anaplastic thyroid cancer cell proliferation in a dose-dependent manner. Roniciclib activated caspase-3 activity and induced apoptosis. Cell cycle progression was arrested in G2/M phase. *In vivo*, the growth of anaplastic thyroid cancer xenograft tumors was retarded by roniciclib treatment without evidence of toxicity. These data provide a rationale for further clinical evaluation using roniciclib in the treatment of patients with anaplastic thyroid cancer.

## INTRODUCTION

Anaplastic thyroid cancer (ATC) is one of the most aggressive human malignancies and is typically lethal. ATC occurs in less than 2% of all thyroid cancers but is responsible for up to 14-39% of the mortality from thyroid cancer [1–3]. ATC is classified as TNM stage IV regardless of tumor burden because of its highly aggressive and lethal nature [4]. Multiple modality treatment, including surgery, external radiotherapy and chemotherapy (including paclitaxel, docetaxel, doxorubicin and cisplatin) is the currently most effective approach to improve local control and survival in patients with ATC confined to the thyroid (stage IVA) or the neck (stage IVB). However, the 2.5-year survival rate is only 50% using such treatment regimens in this subset of patients [5, 6]. There is no effective treatment for metastatic ATC (stage IVC) that consists of approximately 40-60% of all ATC patients at the time of diagnosis [7–9]. Novel therapies with different mechanisms of activity are needed to improve the outcomes of patients with this dismal disease.

Cyclin-dependent kinases (CDKs) are serine/threonine kinases involved in the cell division cycle. Specific CDKs are activated by interacting with their regulatory cyclins. Activation of CDK-cyclin complexes promote cell cycle entry and progression in a regulated manner [10, 11]. The formation of CDK4/6-cyclin D and CDK3-cyclin C complexes contribute to cell cycle entry (G0 to G1 phase). These complexes also promote the expression of multiple cell cycle proteins, including cyclin E. CDK2-cyclin E complex (and to a lesser extent, CDK1/3-cyclin E) drives G1 to S phase transition. CDK1/2-cyclin A complexes further ensure cell cycle progression through S to G2 phase. Finally, the CDK1-cyclin B1 complex is required for transition from G2 to mitotic entry and progression in mitotic phase.

Altered CDK activity is observed in many human cancer types [12]. In addition to their canonical roles in cell cycle regulation, the CDK family has other important biologic functions, including transcription and control of cell survival [11]. For example, CDK5 is pivotal for neural development and regulates cell proliferation of medullary thyroid cancer [13, 14]. CDK7 is important for the survival of triple-negative breast cancer cells [15]. CDK9 is required for Myc-driven liver tumor maintenance [16]. CDKs are essential to the control of cell proliferation and survival, and the inhibition of CDK activity represents an important therapeutic strategy in the treatment of human malignancies [17].

Roniciclib is a potent pan-CDK inhibitor that inhibits the activity of cell-cycle CDKs (CDK1, CDK2, CDK3, CDK4) with IC_50_ values at low nanomolar range (≤ 11 nM) [18]. In addition to cell-cycle CDKs inhibition, roniciclib also represses the activity of transcriptional CDKs (CDK5, CDK7, CDK9) and non-CDK kinases, including Aurora A, at IC_50_ values ≤ 50 nM. Preclinical studies have demonstrated that roniciclib arrests cell cycle progression, activates caspase-3 activity, and induces apoptosis *in vitro*. Following oral administration in mice, roniciclib is rapidly absorbed and has potent efficacy in inhibiting tumor growth of cervical and lung cancer xenografts with acceptable safety profiles. These data suggest that roniciclib has the potential to treat patients with other types of malignancy as well.

In this study, we evaluated the therapeutic effects of roniciclib on three ATC cell lines using both *in vitro* and *in vivo* assays to assess for clinical applicability.

## RESULTS

### Roniciclib induced cytotoxicity in ATC cell lines

**Figure 1:**
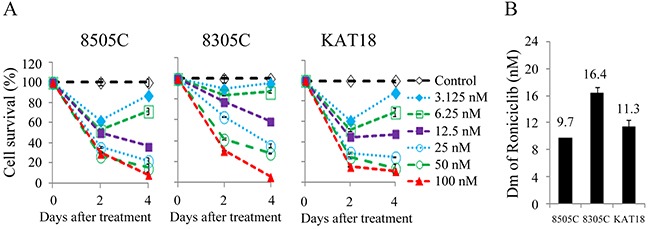
Roniciclib induces cytotoxicity in ATC cells **(A)** Cytotoxicity was evaluated in cells treated with a series of six 1:1 dilutions of roniciclib. Dose-response curves were obtained on day 2 and day 4 using LDH assay. **(B)** Median-effect dose (Dm) of roniciclib on day 4 was calculated for each cell line using CompuSyn software.

Roniciclib inhibited cell proliferation in three ATC cell lines in a dose-dependent manner (Figure 1A). All doses studied demonstrated to inhibit cell proliferation. Higher doses of roniciclib (≥ 25 nM) revealed more robust and durable effects to induce cytotoxicity over a 4-day treatment course. Roniciclib at 25 nM inhibited at least 64.2% of cell growth by day 4. At 100 nM, roniciclib arrested > 89.3% cell growth in these ATC lines. The potency of cytotoxicity of roniciclib in ATC cell lines was determined using the CompuSyn software [19, 20]. The median-effect dose (Dm) was determined on day 4 (Figure 1B). 8505C cells had the lowest Dm (9.7 ± 0.1 nM), followed by KAT18 (11.3 ± 1.1 nM) and 8305C (16.4 ± 0.8 nM). These cytotoxic effects of roniciclib in three ATC cell lines were confirmed by counting viable cells under the microscope after a four-day therapy (Supplementary Figure 1).

### Effects of roniciclib on apoptosis

**Figure 2:**
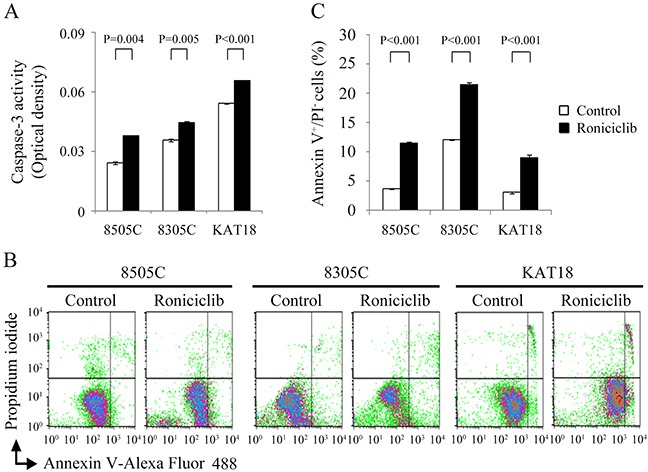
Roniciclib activates caspase-3 activity and induces apoptosis in ATC cells **(A)** Caspase-3 activity was detected using fluorometric assay kit in cells treated with roniciclib (25 nM) or vehicle for 24 h in 8505C, 8305C and KAT18 cells. **(B)** Early apoptotic cells were determined by flow cytometry to detect Annexin V-positive/PI-negative staining in cells treated with roniciclib (25 nM) or vehicle for 24 h. **(C)** Statistical analyses of early apoptotic cells showed roniciclib significantly induced early apoptosis in 8505C, 8305C and KAT18 cells.

Apoptosis is a type of programmed cell death that may be induced pharmacologically in cancer therapy [21]. Roniciclib has previously been demonstrated to activate caspase-3/7 activity and induce apoptosis in a cervical cancer cell line [18]. We evaluated the effects of roniciclib on apoptosis in ATC cell lines. The effects of roniciclib (25 nM) on caspase-3 activity were determined using a fluorometric assay at 24 h in 8505C, 8305C and KAT18 cells (Figure 2A). Roniciclib significantly increased caspase-3 activity when compared with control treatment in 8505C (0.038 ± 0.0002-optical density [OD] and 0.0243 ± 0.0009-OD, P = 0.004), 8305C (0.0447 ± 0.0003-OD and 0.0358 ± 0.0005-OD, P = 0.005) and KAT18 (0.0658 ± 0.0001-OD and 0.0542 ± 0.0002-OD, P < 0.001), demonstrating activation of caspase-3. Activation of caspase-3, a major executioner caspase, may contribute to apoptotic cell death. The effect of roniciclib (25 nM) on early apoptosis was evaluated using Annexin V-Alexa Fluor 488 and propidium iodide (PI) staining at 24 h in three ATC cell lines (Figure 2B). Statistical analyses reveal roniciclib significantly increased early apoptotic cells when compared with placebo therapy in 8505C (11.5 ± 0.1% and 3.7 ± 0.1%, P < 0.001), 8305C (21.4 ± 0.5% and 12.0 ± 0.1%, P < 0.001) and KAT18 (9.0 ± 0.4% and 3.1 ± 0.2%, P < 0.001), indicating induction of apoptosis (Figure 2C).

### Effects of roniciclib on cell cycle

**Figure 3:**
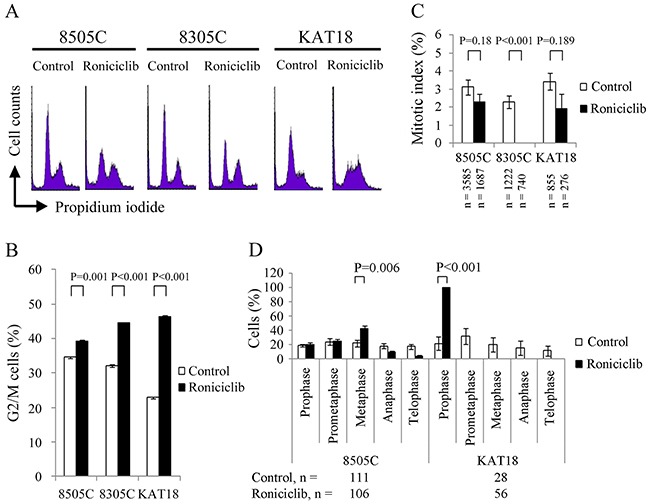
Roniciclib accumulates cells in G2/M phase and inhibits mitotic progression **(A)** Cell cycle analysis was performed to evaluate the DNA content using flow cytometry in 8505C, 8305C and KAT18 cells treated with placebo or roniciclib (25 nM) for 24 h. **(B)** Quantification analyses revealed that roniciclib (25 nM) significantly arrested cells in G2/M phase at 24 h in all three ATC cell lines. **(C)** The percentage of ATC cells in mitosis was assessed after treatment with placebo or roniciclib (25 nM) for 24 h. Cells were stained with DAPI and chromosome characteristics were evaluated using immunofluorescence confocal microscopy. Mitotic index was assessed with a minimum of 276 cells counted from at least ten different fields for each condition. Roniciclib significantly decreased the proportion of cells in mitosis in 8305C cell line. **(D)** The distribution of cells in mitosis was evaluated by counting a minimum of 28 mitotic cells by confocal microscopy for each condition in 8505C and KAT18 cell lines. Statistical analyses revealed roniciclib (25 nM for 24 h) significantly accumulated 8505C cells in metaphase and KAT18 cells in prophase.

Roniciclib has demonstrated the ability to accumulate synchronously growing HeLa cervical cancer cells in G2/M phase [18]. The effect of roniciclib (25 nM for 24 h) on cell cycle distribution was evaluated in three ATC cell lines (Figure 3A). The cell cycle data were analyzed (Figure 3B). Compared with control cells, roniciclib significantly induced cell accumulation in G2/M phase in 8505C (39.2 ± 0.5% and 34.5 ± 0.1%, P = 0.001), 8305C (44.5 ± 0.2% and 32.1 ± 0.3%, P < 0.001) and KAT18 (46.4 ± 0.3% and 22.9 ± 0.3%, P < 0.001), demonstrating induction of G2/M arrest.

The ability of roniciclib to accumulate cells in mitotic phase was determined using confocal fluorescence microscope (Supplementary Figure 2). Mitotic cells were identified and mitotic index was calculated for three ATC cell lines (Figure 3C). Compared with control cells, roniciclib (25 nM) treatment for 24 h significantly decreased the percentage of mitotic cells in 8305C (0.0 ± 0.0% and 2.3 ± 0.4%, P < 0.001), and insignificantly decreased the proportion of mitotic cells in 8505C (2.3 ± 0.4% and 3.1 ± 0.4%, P = 0.180) and KAT18 (1.9 ± 0.9% and 3.4 ± 0.5%, P = 0.189), demonstrating that roniciclib inhibited 8305C cells into mitosis.

The distribution of cells in mitosis was evaluated in 8505C and KAT18 cells (Figure 3D). Compared with control treatment, roniciclib (25 nM for 24 h) significantly increased the proportion of metaphase cells in 8505C (42.1 ± 4.3% and 21.8 ± 4.9%, P = 0.006) and prophase cells in KAT18 (100.0 ± 0.0% and 21.7 ± 9.3%, P < 0.001), revealing roniciclib arrested mitotic progression in metaphase (8505C) and prophase (KAT18).

### Roniciclib modulates the expression of aurora a and survivin

**Figure 4:**
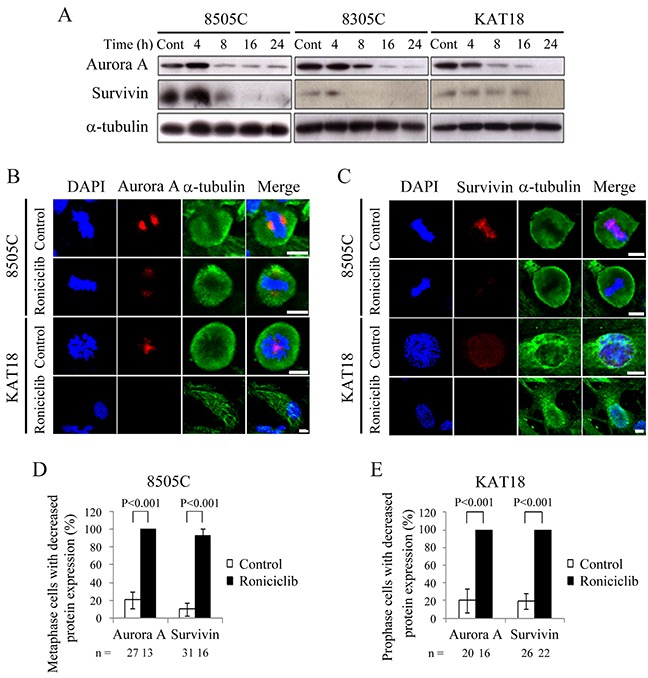
Effects of roniciclib on the expression of Aurora A and survivin in ATC cells **(A)** The expression of Aurora A and survivin was evaluated by Western blotting in 8505C, 8305C and KAT18 cells treated with roniciclib (25 nM) or placebo for the indicated periods. **(B)** Cells were treated with roniciclib (25 nM) or placebo for 24 h and stained with fluorescent antibodies against DAPI (blue), Aurora A (red) and α-tubulin (green). Aurora A level was reduced after treatment of roniciclib in metaphase cells of 8505C and prophase cells of KAT18. **(C)** Cells were treated with roniciclib (25 nM) or placebo for 24 h and stained with fluorescent antibodies against DAPI (blue), survivin (red) and α-tubulin (green). Survivin level was significantly reduced after treatment of roniciclib in metaphase cells of 8505C and prophase cells of KAT18. **(D)** The percentages of 8505C metaphase cells with decreased Aurora A and survivin level were assessed after treatment with placebo or roniciclib (25 nM) for 24 h. Cells were stained with Aurora A and survivin and their expression was evaluated using immunofluorescence confocal microscopy. A minimum of 13 metaphase cells was counted for each condition. Roniciclib significantly increased the proportion of metaphase cells with low Aurora A and survivin levels in 8505C. **(E)** The percentages of KAT18 prophase cells with low Aurora A and survivin level were assessed after treatment with placebo or roniciclib (25 nM) for 24 h. Cells were stained with Aurora A and survivin and their expression was evaluated using immunofluorescence confocal microscopy. A minimum of 16 prophase cells was counted for each condition. Roniciclib significantly increased the proportion of prophase cells with decreased Aurora A and survivin levels in KAT18. Scale bar, 10 μm.

Aurora A is a protein essential for G2/M transition and mitotic progression [22, 23]. Survivin is required for chromosome alignment and chromosome segregation during mitosis [24, 25]. The effects of roniciclib (25 nM) on the expression of these proteins were evaluated in three cell lines (Figure 4A). The ratios of Aurora A and survivin to α-tubulin in each cell line were calculated. Relative expression was calculated using the control value as reference (Supplementary Figure 3). In 8505C and 8305C cells, Aurora A and survivin levels were transiently increased by 4 h, then decreased by 8-16 h with inhibitory effects persisting for 24 h. In KAT18, Aurora A and survivin levels were decreased by 8 h and 24 h, respectively. These results demonstrate that 24-h roniciclib treatment decreased Aurora A and survivin expression in three ATC cell lines. Confocal fluorescence microscopy showed that roniciclib (25 nM for 24 h) decreased Aurora A (Figure 4B) and survivin (Figure 4C) expression in metaphase of 8505C cells and in prophase of KAT18 cells. The percentages of mitotic cells with low expression of Aurora A and survivin were analyzed in 8505C (Figure 4D) and KAT18 (Figure 4E) cell lines. Roniciclib (25 nM for 24 h) significantly increased the proportion of 8505C metaphase cells with decreased expression of Aurora A (100.0 ± 0.0% and 20.5 ± 9.5%, P < 0.001) and survivin (92.9 ± 7.1% and 10.0 ± 7.2%, P < 0.001) when compared with control. Similar observations appeared in KAT18 cells. Roniciclib treatment increased the proportion of KAT18 prophase cells with low level of Aurora A (100.0 ± 0.0% and 20.0 ± 13.3%, P < 0.001) and survivin (100.0 ± 0.0% and 19.2 ± 9.0%, P < 0.001) when compared with control. These results demonstrate that roniciclib diminishes Aurora A and survivin expression in mitotic ATC cells.

### Roniciclib therapy of murine flank tumors

**Figure 5:**
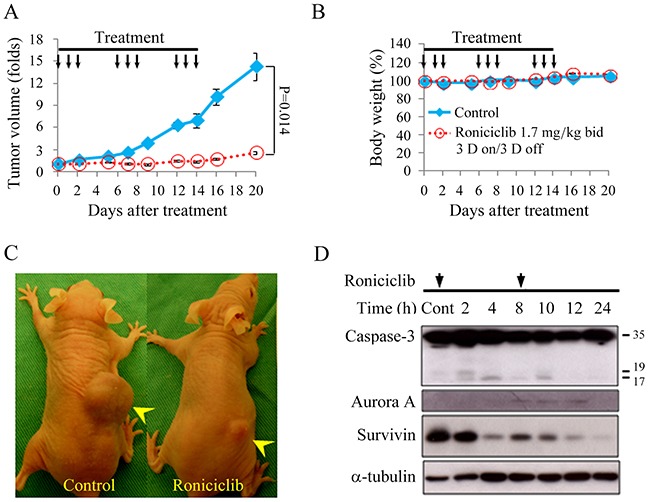
Roniciclib inhibits subcutaneous xenograft growth of 8505C tumor **(A)** The therapeutic effects of roniciclib were evaluated in mice bearing 8505C flank tumors. Serial oral gavage of roniciclib (1.7 mg/kg) significantly repressed 8505C tumor growth after 2 days when compared with control mice. **(B)** Serial treatment of roniciclib did not significantly induce changes in weight when compared with control mice during the study period. **(C)** Photos of representative mice on day 16 of treatment were shown. **(D)** The molecular effects of roniciclib treatment were evaluated in 8505C tumors using Western blot analysis. Arrow, roniciclib and placebo treatment. Arrowhead, 8505C xenograft tumor.

Nude mice bearing flank xenografts of 8505C cells were used to evaluate the therapeutic efficacy and safety of roniciclib *in vivo*. Animals with established flank tumors with a mean diameter of 5.1 mm were treated with oral gavage of placebo (*n* = 5) or roniciclib (*n* = 5) twice a day for three cycles of 3-day on and 3-day off therapy. Administration of roniciclib (1.7 mg/kg) significantly retarded 8505C tumor growth on day 2 as compared with the control group (1.01 ± 0.03-fold and 1.60 ± 0.09-fold, P < 0.001), and the effect persisted through day 20 (2.56 ± 0.45-fold and 14.30 ± 3.33-fold, P = 0.014; Figure 5A). Serial treatment of roniciclib did not result in significant changes in body weight (Figure 5B). These data demonstrate the promising therapeutic efficacy and safety profile of roniciclib treatment *in vivo*. Representative mice were photographed on day 16 of treatment (Figure 5C).

The molecular effects of roniciclib treatment (1.7 mg/kg bid) in 8505C xenografts were evaluated (Figure 5D). The ratios of cleaved caspase-3 (active form of caspase-3), Aurora A and survivin to α-tubulin at each time point were calculated. Relative expression was analyzed using the control value as reference (Supplementary Figure 4). Roniciclib treatment rapidly (by 2 h) increased the level of cleaved caspase-3, though the effect was absent by 8 h. Survivin level was decreased by 4 h and these inhibitory effects persisted for 24 h. Surprisingly, Aurora A level transiently increased between 4 and 12 h, before dropping by 24 h. Collectively, these findings suggest that the increased expression of cleaved caspase-3 and decreased expression of survivin induced by roniciclib led to apoptosis and inhibited cell proliferation in 8505C tumors.

## DISCUSSION

Roniciclib effectively inhibited cell proliferation with a relatively low median-effect dose (≤ 16.4 nM) in three ATC cell lines. Roniciclib effectively represses tumor growth of an ATC xenograft model (8505C) with a promising safety profile, suggesting that this drug has the potential in the treatment of patients with ATC.

Roniciclib treatment led to apoptotic cell death in ATC cell lines. Apoptosis is mediated through activation of extrinsic (death receptor) pathway and intrinsic (mitochondrial) pathway [21]. Multiple CDKs are involved in apoptosis signaling pathways [11]. Inhibition of CDKs activity is therefore likely to contribute to apoptosis. We sought to clarify the underlying mechanisms of roniciclib activation of caspase-3 activity in ATC cells.

Roniciclib accumulated cells in G2/M phase in three ATC cell lines. Roniciclib completely abolished the presence of mitotic cells in 8305C and also led to an insignificant decrease in mitotic cells of 8505C and KAT18. In addition. The fact that mitotic cells were not increased indicate that roniciclib accumulated cells in G2 phase. Thus, G2 phase accumulation is likely one of the mechanisms of cytotoxicity in all three ATC cell lines. In addition to G2 phase arrest, roniciclib inhibited cell cycle progression in metaphase of 8505C cells and in prophase of KAT18 cells, suggesting mitotic arrest is another mechanism contributing to cytotoxicity in 8505C and KAT18 cells.

The decreased levels of Aurora A and survivin may account for cell cycle arrest in G2/M phase in ATC cells. Aurora A is a serine/threonine kinase that is needed for G2/M transition and mitotic progression [22, 23]. Survivin is one of the components of chromosome passenger complex that is pivotal for mitosis [24, 25]. The effects of decreased levels of Aurora A and survivin may range from a failure in G2/M transition to mitotic arrest, depending on the magnitudes of these proteins affected. In this study, roniciclib induced cell accumulation in G2 phase in three cell lines and prevented mitotic progression in the 8505C and KAT18 cell lines.

Survivin is essential for chromosome alignment and segregation during mitosis. Down-regulation of survivin level leads to generates misaligned chromosomes and lagging chromosomes in mitotic cells [24]. We noted that roniciclib (25 nM for 24 h) treatment resulted in higher proportion of mitotic cells with misaligned chromosomes and lagging chromosomes in 8505C cells (Supplementary Figure 5), likely reflecting the effects of survivin depletion.

Roniciclib treatment inhibited 8505C tumor growth. The anti-tumor effects of roniciclib are likely mediated through apoptosis induction and cell cycle inhibition, as cleaved caspase-3 level were found to be increased and survivin levels were decreased after treatment. These effects appeared just 2-4 h following roniciclib administration, suggesting that the therapeutic effects emerge quickly with therapy. These rapid effects may later translate to significant tumor volume differences over 20 days. No significant weight loss was observed in this study, indicating a promising safety profile. Although Aurora A was consistently repressed in ATC cell lines by roniciclib *in vitro*, surprisingly roniciclib treatment *in vivo* transiently increased Aurora A levels in 8505C xenografts before decreasing them at 24 h.

To gain more insights into the therapeutic efficacy of roniciclib in ATC tumors, we evaluated the effect of a treatment regimen of roniciclib (1.7 mg/kg once a day of four-day on and three-day off regimen) in mice bearing 8305C xenograft tumors (Supplementary Figure 6). Serial treatment of roniciclib demonstrated a trend towards slower 8305C tumor growth after 14 days as compared with the control group, but this was not statistically significant.

Paclitaxel has demonstrated a 53% response rate in the treatment of ATC in a phase II trial [26]. The combination effects of roniciclib and paclitaxel against 8505C, 8305C and KAT18 cells were studied (Supplementary Figure 7). The interactions between roniciclib and paclitaxel were evaluated using Chou-Talalay equation. In these cell lines, the best combination effects appeared mostly in 8305C cells and at lower affected fractions.

A flank tumor model of 8305C in nude mice was used to study the effects of roniciclib plus paclitaxel therapy *in vivo* (Supplementary Figure 8). Mice with established flank tumors were treated with vehicle or combination therapy of roniciclib (1.7 mg/kg) p.o. and paclitaxel (20 mg/kg) i.p. once every three days. 8305C flank tumors receiving combination therapy failed to reveal significantly reduced tumor growth as compared with control group during a 6-day treatment period. However, combination therapy significantly induced body weight loss on day 6. These results indicate this combination treatment regimen of roniciclib and paclitaxel did not have therapeutic benefit when side effects appeared.

There are very few clinical trials using roniciclib for the treatment of malignancy. A recent report revealed the addition of roniciclib to platinum-based first line chemotherapy did not improve the therapeutic effects of standard chemotherapy in patients with extensive small cell lung cancer [27]. In addition, higher incidences of adverse event occurred in the roniciclib combination treatment group. However, the efficacy of roniciclib in the treatment of patients with ATC is unknown and needs to be explored.

In conclusion, the CDK inhibitor, roniciclib, induces cytotoxicity in three ATC cell lines. *In vivo* studies using 8505C ATC xenografts demonstrates significant therapeutic efficacy and safety. These data support the planning of evaluating the potential use of roniciclib in the treatment of patients with ATC.

## MATERIALS AND METHODS

### Cell lines

Three human ATC cell lines were evaluated, including 8505C, 8305C and KAT18 [28–31]. All cell lines were authenticated using DNA short tandem repeat profiling and stored in liquid nitrogen until use. 8505C and 8305C were maintained in MEM with sodium pyruvate (1 mmol/L) and sodium bicarbonate (2.2 g/L). KAT18 was maintained in RPMI 1640 with sodium bicarbonate (2.0 g/L). All media contained 10% FCS, 100,000 units/L penicillin and 100 mg/L streptomycin. All cells were maintained in a 5% CO_2_ humidified incubator at 37°C.

### Pharmacologic agents

Roniciclib was a generous gift from Bayer Pharma AG (Berlin, Germany) and was dissolved in DMSO (Sigma) to a concentration of 10 mM and stored at −30°C until further use *in vitro* experiments. For the *in vivo* studies, roniciclib was diluted in vehicle [40% poly(ethylene glycol) 300 (Sigma) and 60% water] to a concentration of 0.357 mg/ml before use.

### Antibodies

Antibodies targeting Aurora A, survivin and caspase-3 were purchased from Cell Signaling Technology. α-tubulin antibody was obtained from Sigma.

### Cytotoxicity assays

Cells were plated at 2 × 10^3^ (8505C, KAT18) or 2 × 10^4^ (8305C) cells per well in 24-well plates in 1 mL media. After an overnight incubation, six serial 1:1 dilutions of roniciclib or vehicle were added at the starting dose of 100 nM over a 4-day treatment course. Cytotoxicity was determined on day 2 and day 4. Culture medium was removed and cells were washed with PBS and lysed with Triton X-100 (1.35%, Sigma) to release intracellular lactate dehydrogenase (LDH), which was quantified with a Cytotox 96 kit (Promega) at 490 nM by spectrophotometry (Infinite M200 PRO, Tecan). Each experiment was performed in triplicate, and the results are shown as the percentage of surviving cells determined by comparing the LDH of each sample relative to control samples, which were considered 100% viable. The median-effect dose (Dm) on day 4 was calculated for each cell line by CompuSyn software using fractions of cell proliferation inhibition [19, 20].

### Apoptosis assessment

Caspase-3 activity was analyzed using fluorometric assay kit (Abcam). Cells were plated at 1 × 10^6^ cells in 100-mm Petri dishes in 10 mL of media overnight. Roniciclib (25 nM) or vehicle was added for 24 h. Adherent cells (5 × 10^5^) were collected, centrifuged, lysed using 50 μL of lysis buffer on ice for 10 min, incubated with DEVD-AFC substrate and reaction buffer at 37°C for 1.5 h. Caspase-3 activity was detected by spectrophotometry. Each condition was performed in duplicate.

Early apoptosis was measured by Annexin V-Alexa Fluor 488 and PI staining kit (Invitrogen). Cells were plated at 2 × 10^5^ (8505C, KAT18) or 3 × 10^5^ (8305C) cells per well in 6-well plates in 2 mL of media overnight and treated with roniciclib (25 nM) or placebo for 24 h. Adherent cells were collected, washed with PBS and incubated with Annexin V-Alexa Fluor 488 and PI at room temperature in the dark for 15 min according to the manufacturer’s protocol. Early apoptotic cells (Annexin V-positive, PI-negative) were detected by flow cytometry (BD FACScalibur Flow Cytometer, BD Biosciences). Each condition was performed in triplicate.

### Cell cycle assessment

The effects of roniciclib on cell cycle progression were evaluated. Cells were plated at 2 × 10^5^ cells per well in 6-well plates in 2 mL of media overnight. Roniciclib (25 nM) or vehicle was added and incubated for 24 h, then both floating and adherent cells were collected, washed with PBS, fixed with cold 70% ethanol and incubated with RNase A (100 μg/mL; Sigma) and PI (5 μg/mL; Sigma) at 37°C for 15 min. Cell cycle distribution was assessed by DNA content detected by flow cytometry (BD FACScalibur Flow Cytometer, BD Biosciences). Each condition was performed in triplicate.

### Immunofluorescence microscopy

The effect of roniciclib on mitotic progression was evaluated using confocal microscopy. Thyroid cancer cells were plated at 5 × 10^4^ cells in four-well culture slides in 1 mL of media overnight. Cells were treated with roniciclib (25 nM) or placebo for 24 h, washed with PBS, fixed in 4% paraformaldehyde (Sigma) for 15 min at room temperature, washed with PBS, permeabilized with 0.1% Triton X-100 (10 min, room temperature), washed with PBS, incubated with 4’,6-diamidino-2-phenylindole (DAPI; 0.2 μg/mL, Invitrogen) for 10 min at room temperature, washed with PBS, and covered with Vectashield mounting medium (Vector Laboratories). Images were captured with Leica TCS SP8 X confocal microscopy (Leica Microsystems). Chromosomes were examined to identify mitotic cells.

The expression of Aurora A and survivin was evaluated using immunofluorescence microscopy. Roniciclib (25 nM) or placebo treated ATC cell samples were prepared as described above. Cells were then incubated with primary rabbit Aurora A antibody (1:200), rabbit survivin antibody (1:200) and mouse α-tubulin antibody (1:1000) at 4°C overnight, washed with PBS and incubated with secondary Alexa Fluor 633-conjugated goat anti-rabbit antibody (1:1000; Invitrogen) and Alexa Fluor 488-conjugated goat anti-mouse antibody (1:1000; Life Technologies) for 25 min at 37°C, washed with PBS, counterstained with DAPI, washed with PBS and covered with mounting medium. Images were acquired using Leica TCS SP8 X confocal microscopy. The expression of Aurora A and survivin was examined in metaphase cells of 8505C and prophase cells of KAT18.

### Western blot analysis

Cells were plated at 1 × 10^6^ cells in 100-mm Petri dishes in 10 mL of media overnight and treated with roniciclib at 25 nM or vehicle for the indicated periods. Cell pellets were dissolved in radio-immunoprecipitation assay buffer and protease inhibitor cocktail, vortexed and clarified by centrifugation. Total protein (20-40 μg) was separated by electrophoresis on 12% Tris-HCl gels, transferred to polyvinylidene difluoride membranes, blocked and exposed to primary antibodies followed by a secondary antibody conjugated to horseradish peroxidase. Signals were developed using an enhanced chemiluminescence kit (PerkinElmer).

### Flank xenograft tumor therapy

Eight-week-old athymic female nude mice from the National Laboratory Animal Center, Taiwan, were anesthetized with an intraperitoneal injection of 2% 2,2,2-tribromoethanol (200 μl/mouse; Sigma) before implantation of ATC cells. 8505C flank tumors were established by injecting 1 × 10^6^ cells in 100 μL of ECM gel (Sigma) into the subcutaneous flanks of nude mice. When 8505C tumors reached 5.1 mm in mean diameter, mice were administered oral gavage of vehicle (*n* = 5) or roniciclib (1.7 mg/kg, *n* = 5) twice a day for three cycles of three-day on and three-day off regimen. This dose was chosen based on a previous report [18]. Tumor dimensions were serially measured with electronic calipers, and the volumes were calculated by the following formula: a x b^2^ x 0.4, where a represents the largest diameter and b is the perpendicular diameter. The body weight of each animal was followed as a marker of toxicity.

Tumor levels of caspase-3, Aurora A and survivin were evaluated in mice treated with oral dosing of roniciclib (1.7 mg/kg) by Western blot analysis. At indicated periods, animals were euthanized with carbon dioxide, and the tumors were harvested, mixed with protein extraction buffer (GE Healthcare), homogenized and sonicated on ice. After centrifugation, clarified supernatants were aliquoted and stored at −80°C for Western blotting.

This study was performed in accordance with the recommendations in the Guide for the Care and Use of Laboratory Animals of the Chang Gung Memorial Hospital, and the protocol was approved by the Committee of Laboratory Animal Center at the Chang Gung Memorial Hospital, Linkou (permission No: 2013121401). Animals were given *ad libitum* access to food and water. The method of euthanasia was CO_2_ exposure for 10 min, at a 20% fill rate of cage volume/min.

### Statistical analyses

Comparisons were performed when appropriate using two-sided Student’s *t* tests (Excel, Microsoft). P < 0.05 was considered statistically significant. Results were expressed as mean ± SE.

## SUPPLEMENTARY MATERIALS FIGURES


