# Supplementary material for: Effects of roniciclib in preclinical models of anaplastic thyroid cancer
Source: Oncotarget. 2017 Jul 8;8(40):67990–8000. doi: 10.18632/oncotarget.19092 (PMC5620230; doi:10.18632/oncotarget.19092)
Supplement: Supplementary file 1 [file oncotarget-08-67990-s001.pdf]

Effects of roniciclib in preclinical models of anaplastic thyroid cancer

SUPPLEMENTARY MATERIALS

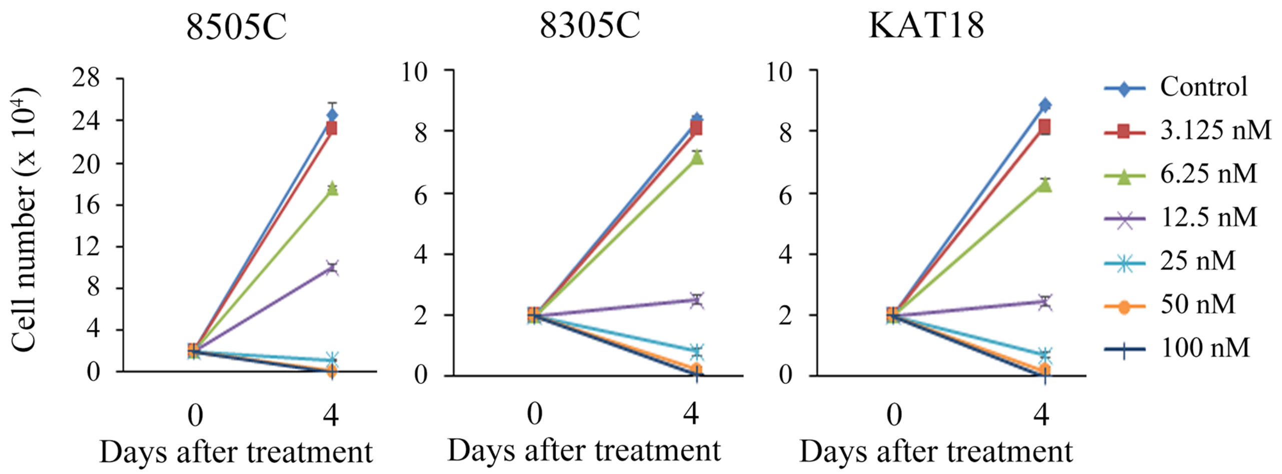

Supplementary Figure 1: Roniciclib inhibits cell proliferation in ATC cell lines. 8505C, 8305C and KAT18 cells were plated at 2 x 10^4 cells per well in 24-well plates in 1 mL media. After an overnight incubation, six serial 1:1 dilutions of roniciclib or vehicle were added at the starting dose of 100 nM. Viable cells were counted under the microscope after a four-day therapy.

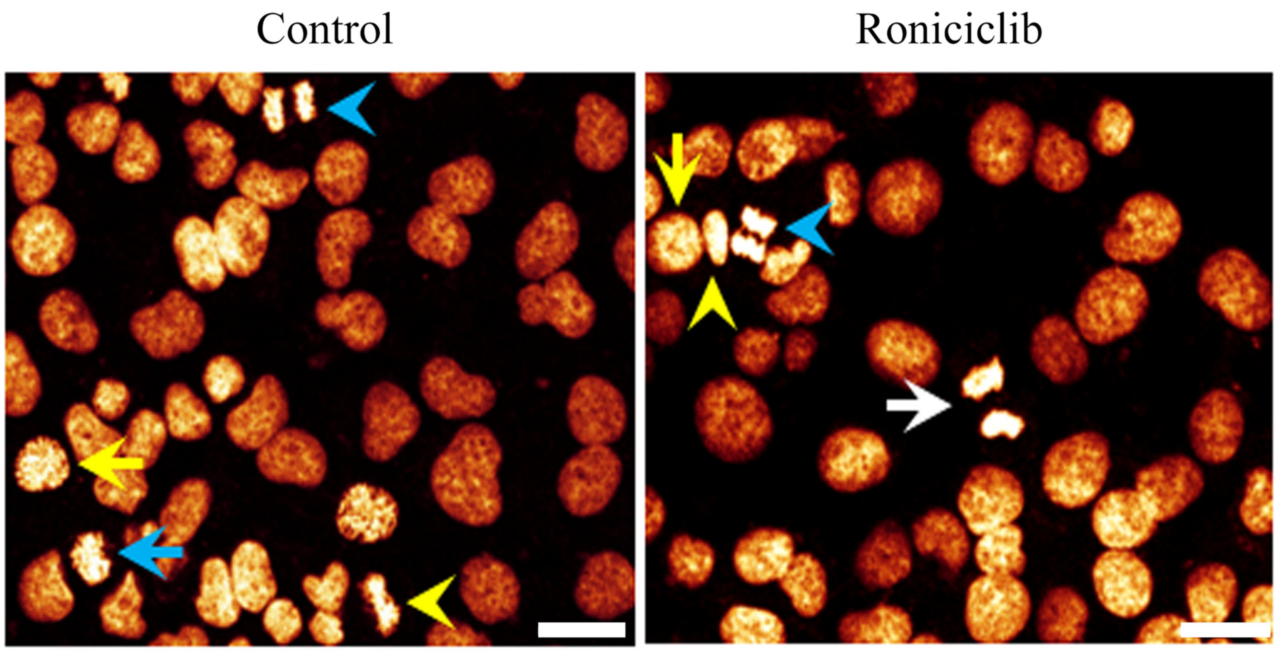

Supplementary Figure 2: The effect of roniciclib on mitosis in 8505C cells. Chromosomal appearance was evaluated in 8505C cells treated with roniciclib (25 nM) or placebo for 24 h using immunofluorescence confocal microscopy. DNA was stained with DAPI. Placebo-treated cells in prophase (yellow arrow), prometaphase (blue arrow), metaphase (yellow arrowhead), and anaphase (blue arrowhead) were indicated. Roniciclib-treated cells in prophase (yellow arrow), metaphase (yellow arrowhead), anaphase (blue arrowhead) and telophase (white arrow) were demonstrated. Scale bar, 10 μm.

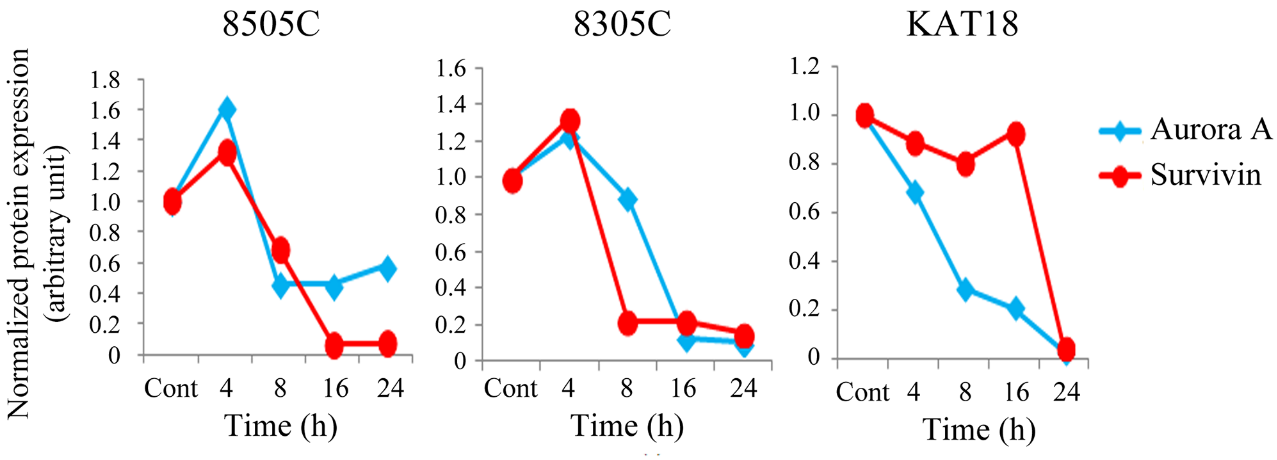

Supplementary Figure 3: Effects of roniciclib on the expression of Aurora A and survivin in ATC cell lines. Roniciclib (25 nM) treatment transiently increased Aurora A and survivin levels by 4 h, then decreased their expression by 8-16 h in 8505C and 8305C cells. Aurora A and survivin levels were decreased by 8 h and 24 h, respectively in KAT18 cells.

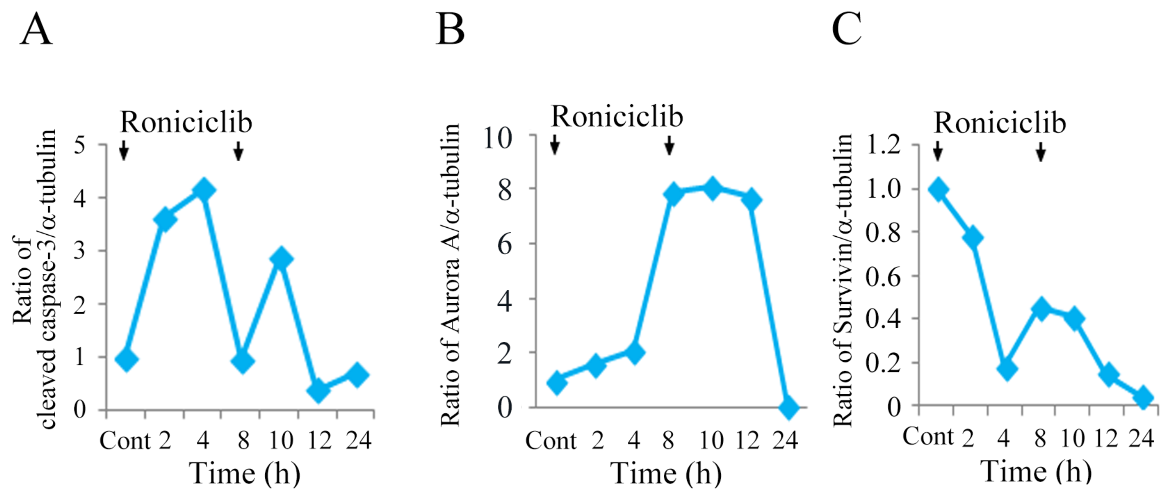

Supplementary Figure 4: Effects of roniciclib on the expression of caspase-3, Aurora A and survivin in 8505C xenograft. **(A)** Oral administration of roniciclib (1.7 mg/kg) increased cleaved caspase-3 level by 2 h after treatment. **(B)** Roniciclib increased Aurora A level by 4 h and the effect persisted till 12 h. **(C)** Survivin level was decreased between 4-24 h. Arrow, roniciclib treatment.

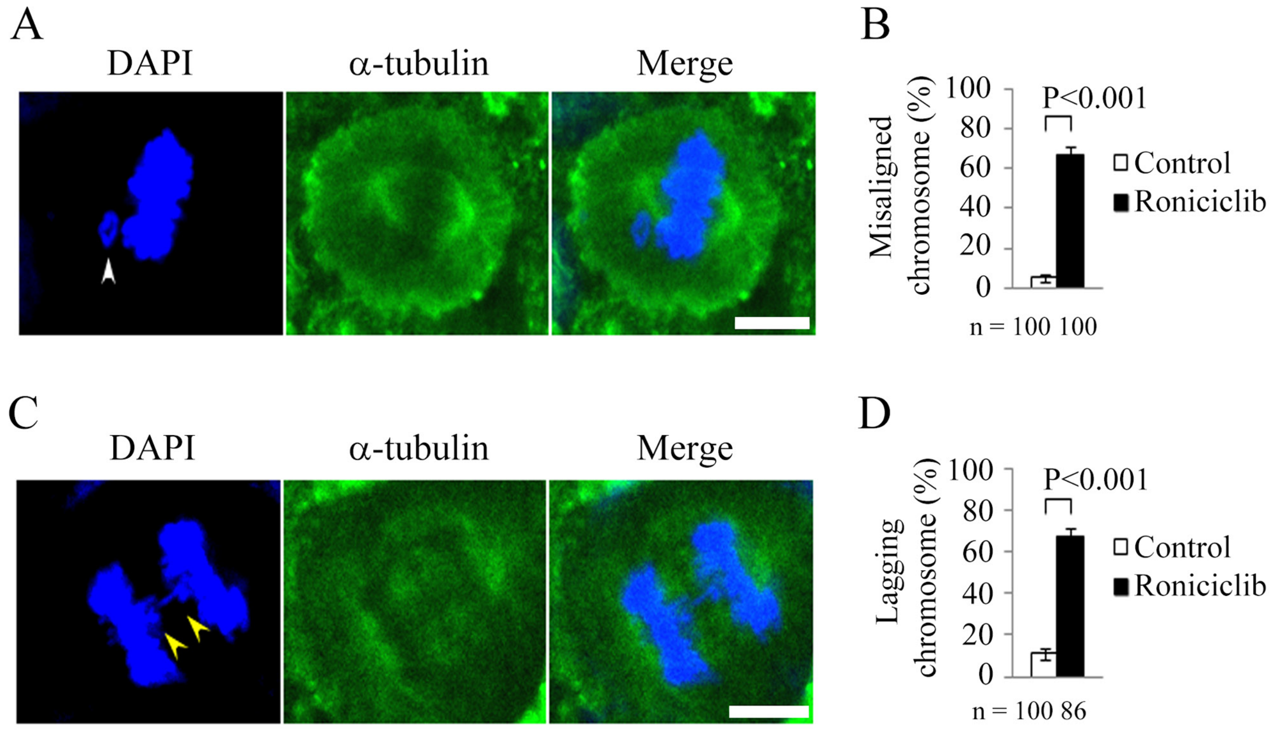

Supplementary Figure 5: Roniciclib increased misaligned chromosomes and lagging chromosomes in mitotic cells of 8505C. **(A)** Cells were treated with roniciclib (25 nM) for 24 h and stained with fluorescent antibodies against DAPI (blue) and α-tubulin (green). A cell in metaphase with misaligned chromosome (white arrowhead) was identified. **(B)** Statistical analysis revealed that roniciclib significantly increased the proportion of metaphase cells with misaligned chromosomes when 100 metaphase cells were counted for each condition. **(C)** Cells were treated with roniciclib (25 nM) for 24 h and stained with fluorescent antibodies against DAPI (blue) and α-tubulin (green). A cell in anaphase with lagging chromosomes (yellow arrowheads) was indicated. **(D)** Quantification analysis revealed roniciclib significantly increased the proportion of anaphase cells with lagging chromosomes when at least 86 anaphase cells were counted for each condition. Scale bar, 10 µm.

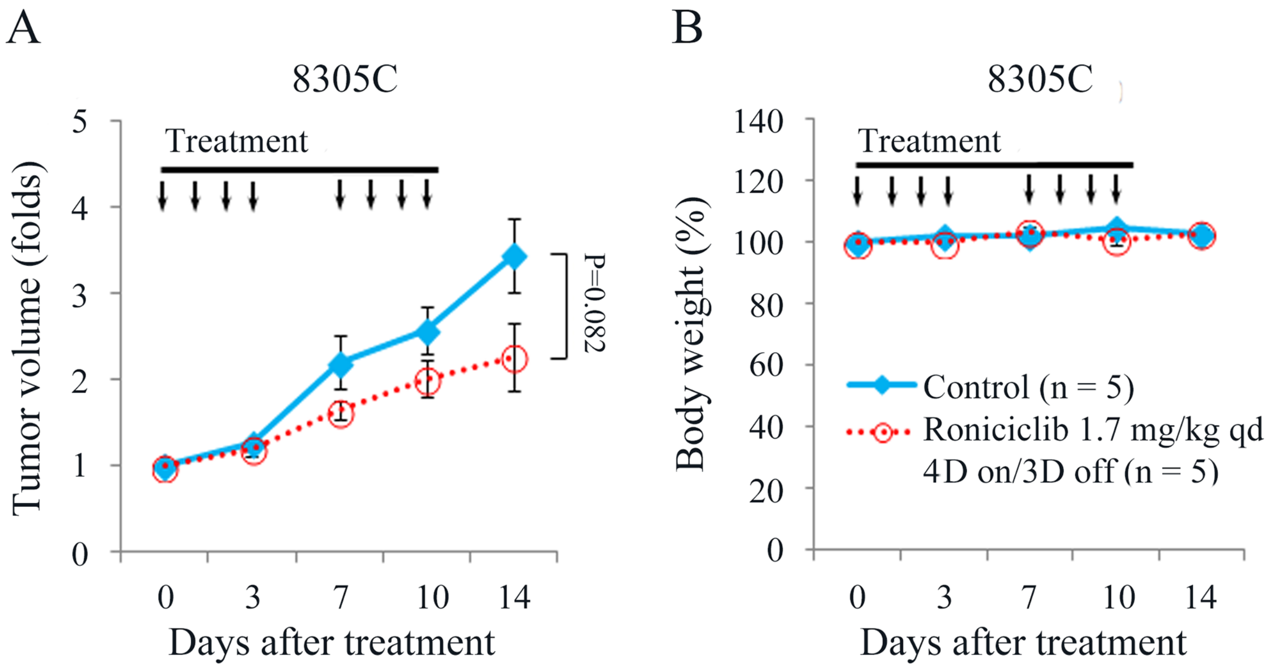

Supplementary Figure 6: The effect of roniciclib on xenograft growth of 8305C tumor. **(A)** Serial treatment of roniciclib (1.7 mg/kg) once a day of two cycles of four-day on and three-day off insignificantly retarded 8305C tumor growth as compared with the control group. **(B)** Sequential treatment of roniciclib did not result in significant changes in body weight. Arrow, roniciclib and placebo treatment.

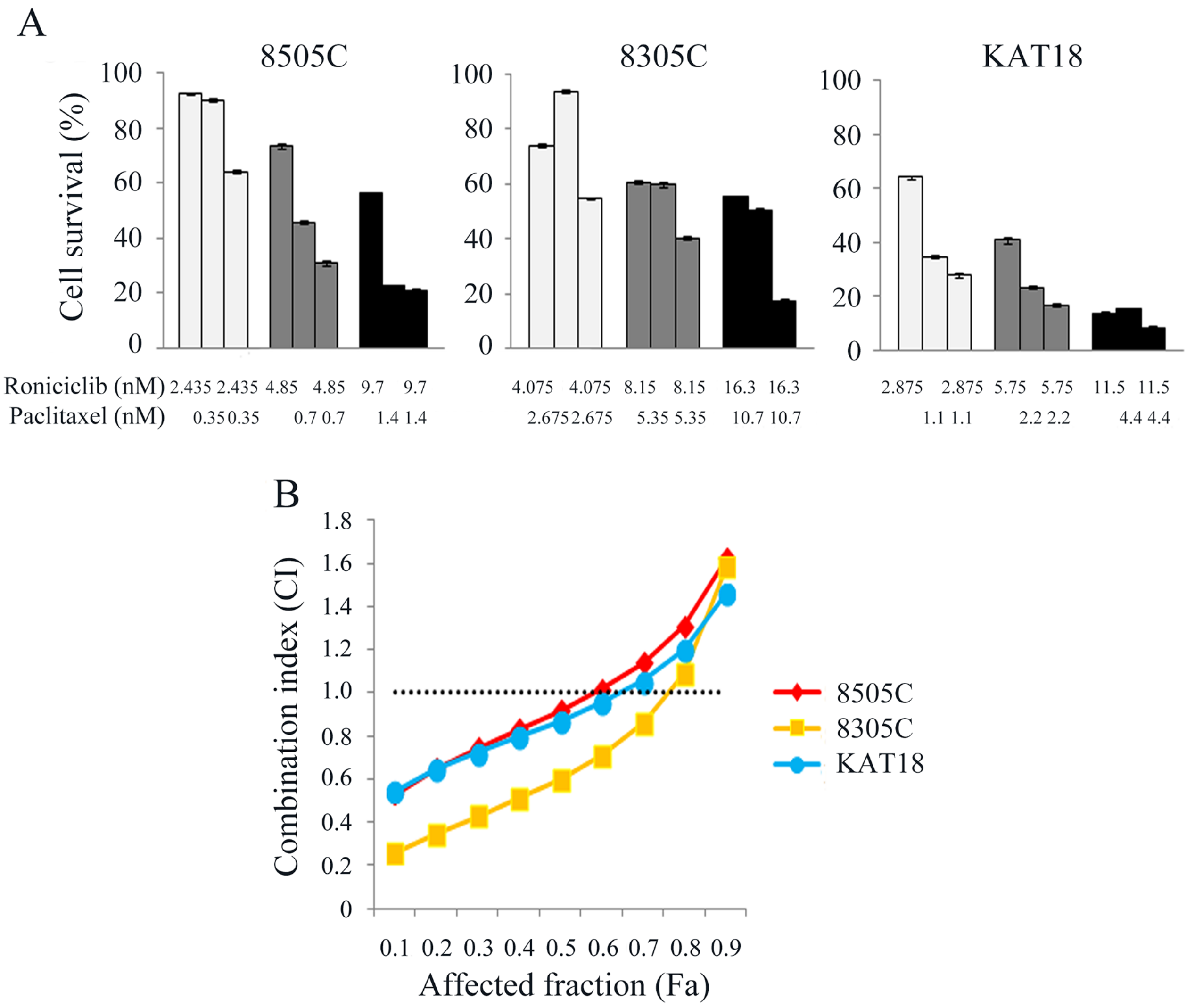

Supplementary Figure 7: The combination effects of roniciclib and paclitaxel in ATC cell lines. **(A)** The cytotoxic effects of roniciclib and paclitaxel alone or in combination after a 4-day treatment in ATC cells were evaluated using LDH assays. **(B)** The combination index (CI) between roniciclib and paclitaxel was calculated using CompuSyn software and plotted against the affected fraction (Fa). CI < 1.0 indicates synergy, CI = 1.0 is addictive, and CI >1.0 indicates antagonism. The horizontal dotted line at CI = 1 was drawn.

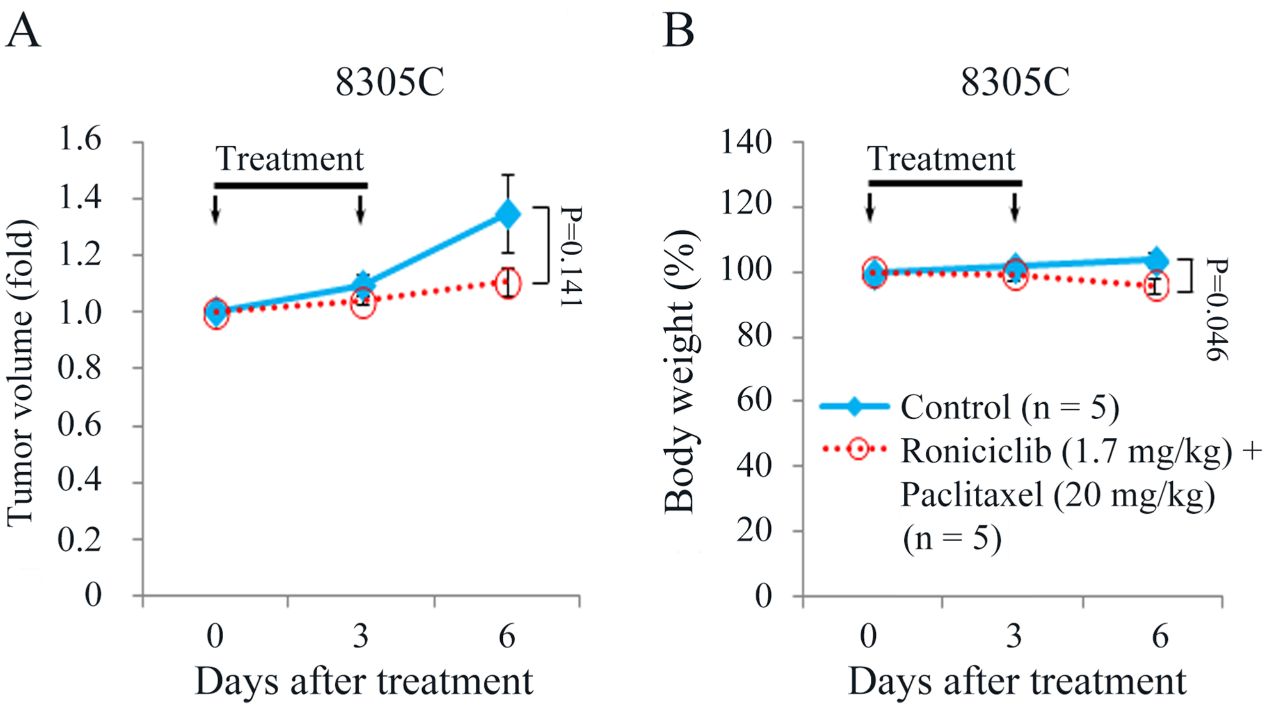

Supplementary Figure 8: Combination effects of roniciclib and paclitaxel on 8305C xenograft tumors. **(A)** Efficacy of serial treatment of combined oral roniciclib and intraperitoneal paclitaxel injections once every three days was evaluated in mice bearing 8305C flank tumors. Combination therapy did not significantly inhibit tumor growth as compared with control during a 6-day treatment period. **(B)** Combination therapy significantly induced body weight loss on day 6. Arrow, placebo and combination treatment.
